# Basis of Shoulder Nerve Entrapment Syndrome: An Ultrasonographic Study Exploring Factors Influencing Cross-Sectional Area of the Suprascapular Nerve

**DOI:** 10.3389/fneur.2018.00902

**Published:** 2018-10-23

**Authors:** Wei-Ting Wu, Ke-Vin Chang, Kamal Mezian, Ondřej Naňka, Chih-Peng Lin, Levent Özçakar

**Affiliations:** ^1^Department of Physical Medicine and Rehabilitation, National Taiwan University Hospital, Bei-Hu Branch and National Taiwan University College of Medicine, Taipei, Taiwan; ^2^Department of Rehabilitation Medicine, First Faculty of Medicine, Charles University in Prague, Prague, Czechia; ^3^First Faculty of Medicine, Institute of Anatomy, Charles University in Prague, Prague, Czechia; ^4^Department of Anesthesiology, National Taiwan University Hospital, Taipei, Taiwan and National Taiwan University College of Medicine, Taipei, Taiwan; ^5^Department of Physical and Rehabilitation Medicine, Hacettepe University Medical School, Ankara, Turkey

**Keywords:** suprascapular nerve, cervical root, sonography, shoulder pain, entrapment neuropathy

## Abstract

As changes in nerves' shape and size are common ultrasonographic findings of entrapment neuropathy, measurement of the nerve cross-sectional area (CSA) becomes the mostly used indicator to differentiate normality from pathology. Recently, more US research has been conducted to measure the shape of the suprascapular notch and the diameter of the suprascapular nerve. Because the suprascapular nerve is paramount for various shoulder disorders, the present study aims to establish normal values of suprascapular nerve sizes at different levels as well as to investigate potential influence of participants' characteristics on the CSA measurements. The present study used a cross-sectional design investigating the CSA values of the suprascapular nerve from the supraclavicular region to spinoglenoid notch. We employed the inside-epineurium and outside-epineurium methods to quantify CSA of cervical roots (C5 and C6) and the suprascapular nerve on US imaging. Univariate comparisons of nerve sizes among different age and gender groups were carried out. Multivariate analysis was performed to analyze the impact of participants' characteristics on nerve CSA. Repeated measurement analysis of variance was conducted to examine segmental variations of CSA of the suprascapular nerve from its origin to infraspinatus fossa. Our study included 60 healthy adults with 120 shoulders and had three major findings: (1) the inside-epineurium method was more reliable than the outside-epineurium approach for CSA measurements due to higher intra- and inter-rater reliability, (2) women had smaller sizes for cervical nerve roots and for the most proximal segment of the suprascapular nerves, and (3) using the outside-epineurium method, the suprascapular nerve CSA was larger in its distal division than the portion proximal to the mid-clavicular line. In conclusion, the inside-epineurium method has better reliability for nerve CSA assessment but the outside-epineurium method is needed for quantifying the size of distal suprascapular nerve. Gender difference in CSA values should be considered during evaluation of the most proximal nerve segment. Using the outside-epineurium method, the distal suprascapular nerve would be estimated larger than its proximal portion and the segmental discrepancy should be not misinterpreted as pathology.

## Introduction

High resolution ultrasound (US) has emerged as a useful tool in the evaluation of nerve entrapment syndromes (1, 2). Complementary to the neurophysiological tests, US is capable of delineating the size and morphology of the diseased nerves and abnormalities in the surrounding structures (3). When the peripheral nerve is entrapped, US imaging can reveal nerve flattening at the compressed site and swollen nerve fascicles which are proximal to the level of compression (4). Changes in the nerve's shape and size are common sonographic findings of entrapment neuropathy, and measurement of the nerve's cross-sectional area (CSA) is the most commonly employed indicator to differentiate between normality and pathology (5). A recent meta-analysis indicated that a cut-off value ranging from 9.0 to 12.6 mm^2^ of the median nerve CSA at the inlet level was a suitable indicator of carpal tunnel syndrome (6). Another meta-analysis showed that ulnar nerve CSA being larger than 10 mm^2^ at the medial epicondyle level could be considered as appropriate criteria to diagnose cubital tunnel syndrome (5). Although there are several quantitative US parameters proposed for evaluation of nerve entrapment syndromes, such as hypoechoic fraction and flattening ratio of the target nerve, few of these can demonstrate diagnostic performance similar to nerve CSA.

The suprascapular nerve innervates the supraspinatus and infraspinatus muscles and provides ~70% of the sensory innervation to the glenohumeral joint (7). High resolution US has been used mostly for assisting intervention of the suprascapular nerve and recent meta-analyses have demonstrated higher consistency and improved effectiveness of the ultrasound guided approach in contrast to the landmark technique in relieving chronic and post-operative shoulder pain (8, 9). Previously, the diagnostic application of US in suprascapular nerve pathology was mostly limited to scrutinizing space occupying lesions, like the paralabral cyst and engorged suprascapular vessels. Recently, more US research has focused on the shape of the suprascapular notch and the diameter of the suprascapular nerve (10–12). The study performed by Gruber et al. proposed a swollen suprascapular nerve as a simple surrogate marker for neuralgic amyotrophy (13). However, the main barrier in employing nerve CSA to diagnose suprascapular neuropathy is the absence of reference values for different age and gender groups. An antecedent study demonstrated that males and tall people were likely to have larger sized median and ulnar nerves (14). As the suprascapular nerve is involved in various shoulder disorders, this study aims to establish the normal reference values of its size at different anatomical regions and for comparisons with the pathological ones and also to investigate the impact of subject characteristics on CSA of the suprascapular nerve.

## Materials and methods

### Participants

This study employed a cross-sectional design to investigate the size of the suprascapular nerve from the supraclavicular region to the infraspinatus fossa. The target population comprised of adults aged over 20 years without any complaint of shoulder discomfort. As the study attempted to explore impact of age, gender, and body status on the nerve size, a total of 60 people were recruited with 10 in each of the 6 defined subgroups. The stratification of the subgroups was based upon differences in sex and age (≥20 to <40 years, ≥40 to <60 years and ≥60 years). The study protocol (20180405RIND) was approved by the institutional review board of National Taiwan University Hospital and all the participants were asked to submit their informed consent before enrollment in the study. The exclusion criteria included shoulder pain, limited shoulder motion, previous shoulder surgery or suprascapular nerve block, and a history of malignancy and rheumatic diseases (e.g., systemic lupus nephritis, ankylosing spondylitis, and rheumatoid arthritis).

### Ultrasound scanning protocol

**Figure 1:**
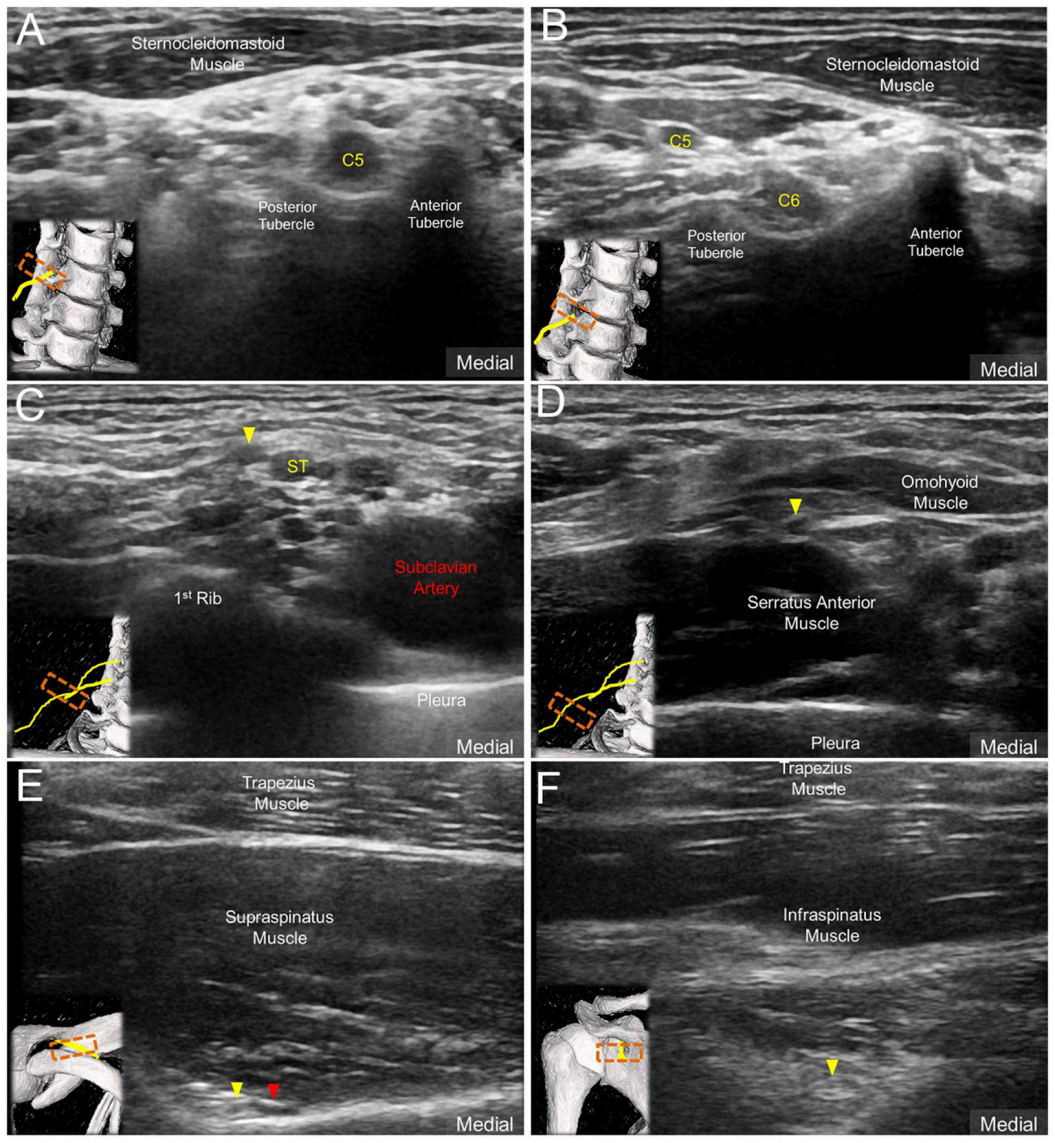
Ultrasound imaging of **(A)** C5 nerve root, **(B)** C6 nerve root and the suprascapular nerve *(solid yellow arrowhead)* **(C)** departing from the superior trunk (ST) of the brachial plexus, **(D)** at the mid-clavicular level, **(E)** inside the supraspinatus fossa, and **(F)** at the spinoglenoid notch of the infraspinatus fossa. Red arrowhead: suprascapular artery.

All examinations were conducted by a musculoskeletal ultrasound specialist with 10 years of experience. Images were obtained using a linear probe of 5–18 MHz (HI VISION Ascendus; Hitachi). The subjects were seated with both arms naturally placed beside the trunk during the examination. Initially, a scout investigation in compliance with the EURO-MUSCULUS/USPRM shoulder protocol (15), including the long head of the biceps tendon, subscapularis tendon, acromioclavicular joint, supraspinatus tendon, infraspinatus tendon, and posterior glenohumeral joint, was performed. Later, the transducer was placed in the horizontal plane at the anterior lateral neck to obtain the images of C5, C6, and C7 nerve roots (Figure 1) (16). The C7 transverse process was first located, which was characterized by a single posterior tubercle without an anterior tubercle. The C7 nerve root was interposed between the posterior tubercle and pulsating vertebral artery. The transducer was then relocated cranially to visualize the C6 nerve exiting the C6 intertubercular groove, formed by its anterior and posterior tubercles (Figure 1A). Likewise, the C5 nerve root could be seen coursing inside the C5 intertubercular groove (Figure 1B).

**Figure 2:**
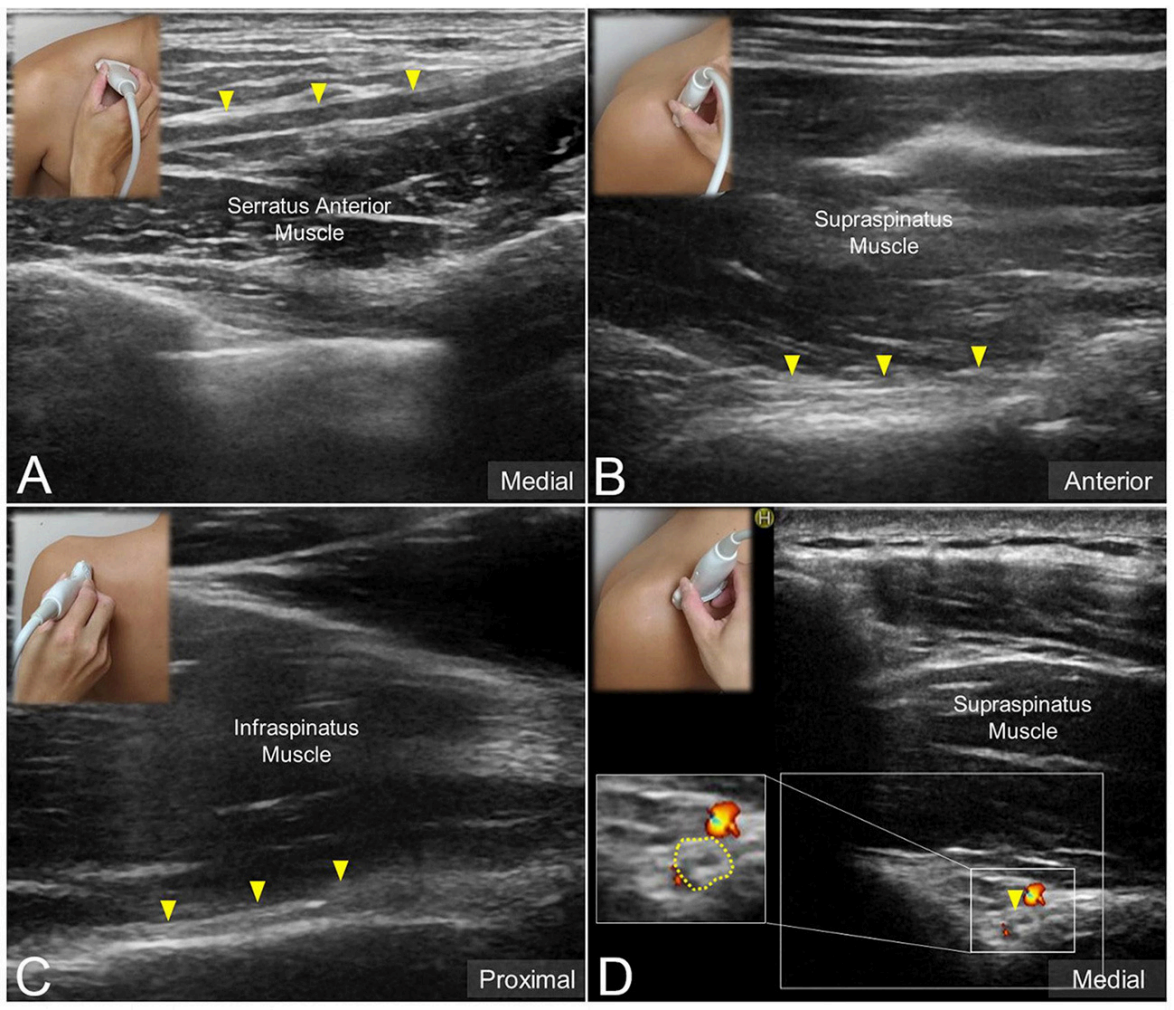
Ultrasound imaging of the suprascapular nerve *(solid yellow arrowhead)* in the long axis at **(A)** the supraclavicular region, **(B)** the supraspinatus fossa, and **(C)** the infraspinatus fossa. Ultrasound Doppler imaging of the suprascapular nerve at the supraspinatus fossa **(D)**. Dashed line: the border of the nerve sheath.

**Figure 3:**
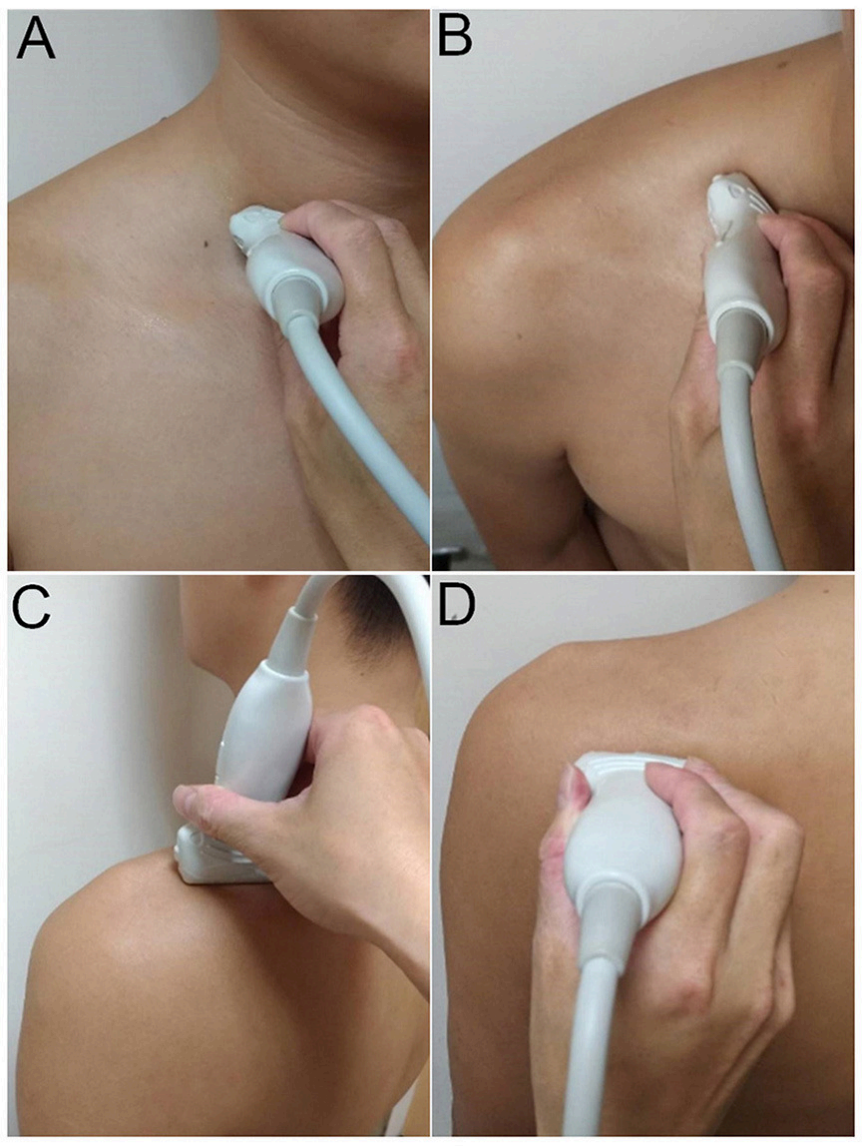
Placement of the ultrasound transducer on the participants for visualization of cervical nerve roots **(A)** and the suprascapular nerve at the supraclavicular region **(B)**, inside the supraspinatus fossa **(C)**, and at the spinoglenoid notch of the infraspinatus fossa **(D)**.

**Figure 4:**
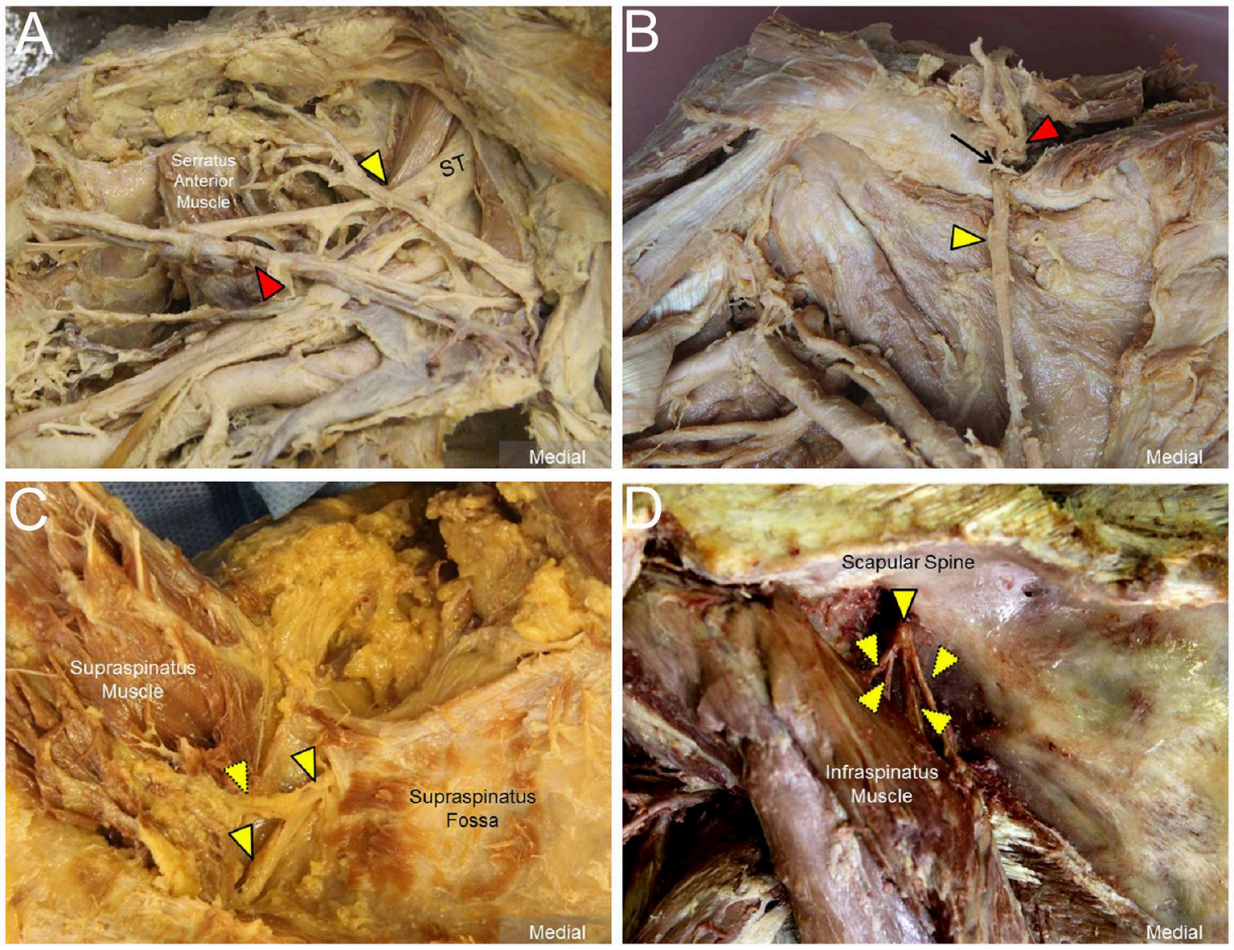
Cadaver shoulder model for the suprascapular nerve *(solid yellow arrowheads)* **(A)** departing from the superior trunk (ST) of the brachial plexus, **(B)** entering the supraspinatus fossa underneath the transverse scapular ligament *(black arrow)*, **(C)** inside the supraspinatus fossa with perineural fat seen surrounding the nerve, and **(D)** at the spinoglenoid notch of the infraspinatus fossa. Red arrowheads: suprascapular artery; dashed yellow arrowheads: branches of the suprascapular nerve.

The transducer was then put along the sagittal plane at the medial edge of the supraclavicular fossa to visualize the supraclavicular brachial plexus (17). The transducer was moved up toward the acromion and the suprascapular nerve was localized as departing from the superior trunk (18) (Figure 1C). Relocating the transducer laterally, the suprascapular nerve was seen at the mid-clavicular level underneath the omohyoid muscle (Figure 1D, and Supplementary Video). The transducer was then redirected to the scapular plane to target the suprascapular nerve in the supraspinatus fossa (Figure 1E). Finally, the transducer was placed along the inferior border of the scapular spine to scan the suprascapular nerve at the spinoglenoid notch (Figure 1F). We also redirected the transducer to align with the long axis of the suprascapular nerve to make sure that the target we visualized was a nerve instead of a random hypoechoic round structure (Figures 2A–C). Power Doppler imaging was also employed to distinguish the accompanying suprascapular vessels in order to exclude them from the CSA measurements (Figure 2D). How the transducer was placed on the participants was shown in Figure 3. The anatomy of the suprascapular nerve was also elaborated using the cadaver shoulder model with the approval of the Anatomical Donation Department of Charles University in Prague (Figure 4).

### Outcome measurement

**Figure 5:**
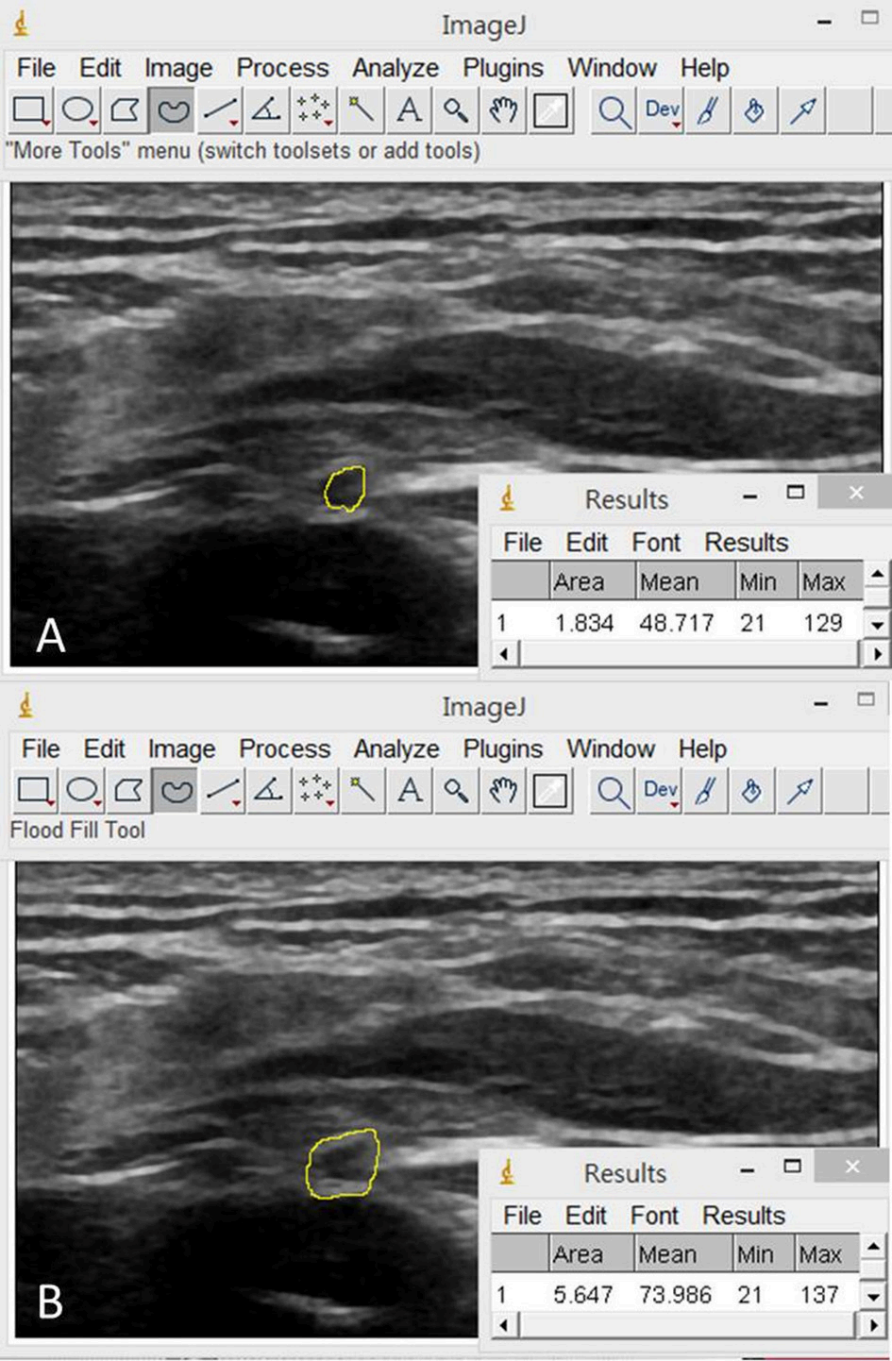
Illustration of the inside-epineurium **(A)** and outside-epineurium **(B)** methods for measurement of the nerve cross-sectional area by using the image processing software, Image J.

The image processing software, Image J (19), was employed for the CSA measurements of the C5 and C6 nerve roots and the suprascapular nerve departing from the upper trunk, at the mid-clavicular line under the omohyoid muscle, inside the supraspinatus fossa and at the spinoglenoid notch of the infraspinatus fossa. We employed two methods to define the border of the target neural structures: inside-epineurium (20) (Figure 5A) and outside-epineurium of the nerve (21) (Figure 5B). Imaged with a high-resolution US transducer, the epineurium appears as a hyperechoic rim surrounding the hypoechoic nerve fascicles. As the nerve fascicles of the suprascapular nerve were difficult to visualize inside the supraspinatus fossa and at the spinoglenoid notch, only outside-epineurium measurements were performed at these sites. Another reason was that the resolution of the ultrasound images was not adequate to differentiate the nerve fascicle from its epineurium at its distal segment due to a decreased transducer frequency for improving sound beam penetration in deeper regions. Using the outside-epineurium method, we circled the outmost circumference of the target nerve for calculation of its CSA. A potential benefit of measuring the CSA along the outer border of the nerve sheath is its less influence by the anisotropic effect.

In addition, we did not measure the suprascapular nerve where it passes underneath the transverse scapular ligament, which is known to be the most common entrapment site. The primary reason was that the nerve courses angularly around the overlying ligament, which rendered the nerve to be anisotropic and difficult to measure.

Before the study was formally initiated, the principal investigator examined 10 shoulders from 5 adults twice, at a 7-day interval, to evaluate intra-observer reliability. A different investigator scanned both shoulders from the same subjects at half a day post the first examination to evaluate inter-rater reliability. Both values were reported using intra-class correlation coefficient (ICC).

### Statistical analysis

The continuous variables were reported using mean and standard deviation (SD), and the categorical data was reported as absolute numbers and percentages. The proportional difference of categorical variables was analyzed by the Chi-square test. Fisher's exact test was employed in case of sparse data distribution. The analysis of variance (ANOVA) was used for comparison of age, body height, body weight, and CSA across various age and sex groups. The Bonferroni procedure was employed for *post-hoc* analysis of CSA values. The generalized estimating equation (GEE) was used to analyze the impact of age, gender, laterality, and body status on the measurements of neural structures. GEE is suitable for dealing with the clustered or correlated data, like the CSA of the right and left suprascapular nerves on the same participants (22). The dependent variables can be scale, counts, binary, or events-in-trials. In the GEE model, the participants' identification was treated as the clustering variable, whereas the laterality (right/left) served as an exchangeable correlation structure. In order to compare the CSA of the suprascapular nerve at 4 different sites, repeated measures ANOVA was used as the size distribution along the same nerve was highly correlated. All of the analyses were performed using SPSS 12.0 and values with *p* < 0.05 were considered to be statistically significant.

## Results

### Basic characteristics of the participants

This study included 60 healthy participants. Male participants had higher average body heights and weights compared to similar aged female participants. In terms of asymptomatic shoulder pathology, the US examination revealed a minimal number of cases with subscapularis tendon calcification, supraspinatus tendon calcification and supraspinatus tendon tears. There was no significant difference in the proportion of pathological findings across the subgroups (Table 1).

**Table 1:** Participants' characteristics and sonographic findings of the examined shoulders.

	**Age** ≥ **20 and** < **40**		**Age** ≥ **40 and** < **60**		**Age** ≥ **60**	
	**Men (10 people/20 shoulders)**	**Women (10 people/20 shoulders)**	**Men (10 people/20 shoulders)**	**Women (10 people/20 shoulders)**	**Men (10 people/20 shoulders)**	**Women (10 people/20 shoulders)**	***p*-value (overall)**
**PARTICIPANTS' CHARACTERISTICS**							
Age (year)	31.4 ± 5.8	33.8 ± 4.5	47.9 ± 5.7	49.6 ± 5.9	69.0 ± 6.2	69.7 ± 6.5	<0.001
Height (cm)	170.6 ± 6.4	160.2 ± 5.4	171.7 ± 5.7	159.6 ± 3.8	166.1 ± 6.0	166.1 ± 6.0	<0.001
Weight (kg)	66.9 ± 5.5	55.1 ± 8.2	70.2 ± 6.7	57.5 ± 9.1	64.9 ± 8.2	64.9 ± 8.2	<0.001
**SHOULDER PATHOLOGY (*****n*****, PERCENT IN SUBGROUPS)**							
Biceps tendinopathy	0 (0%)	0 (0%)	0 (0%)	0 (0%)	0 (0%)	0 (0%)	N.A.
Subscapularis tendinopathy	0 (0%)	0 (0%)	0 (0%)	0 (0%)	0 (0%)	0 (0%)	N.A.
Subscapularis calcification	0 (0%)	0 (0%)	0 (0%)	1 (5%)	0 (0%)	0 (0%)	0.411
Supraspinatus tendinopathy	0 (0%)	0 (0%)	0 (0%)	0 (0%)	0 (0%)	0 (0%)	N.A.
Supraspinatus calcification	0 (0%)	0 (0%)	0 (0%)	0 (0%)	1 (5%)	0 (0%)	0.411
Supraspinatus tendon tear	0 (0%)	0 (0%)	0 (0%)	1 (5%)	1 (5%)	0 (0%)	0.540
Infraspinatus tendinopathy	0 (0%)	0 (0%)	0 (0%)	0 (0%)	0 (0%)	0 (0%)	N.A.
Infraspinatus calcification	0 (0%)	0 (0%)	0 (0%)	0 (0%)	0 (0%)	0 (0%)	N.A.
Infraspinatus tendon tear	0 (0%)	0 (0%)	0 (0%)	0 (0%)	0 (0%)	0 (0%)	N.A.

***Annotation**: N.A, not applicable due to zero count in each selected cell*.

### Reliability of US measurements for nerve CSA

**Figure 6:**
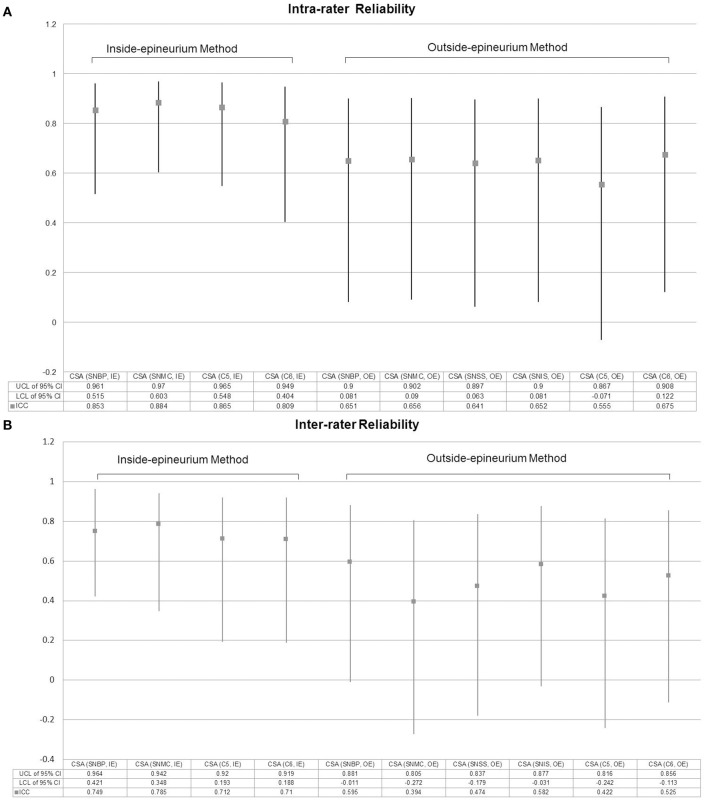
Intra-rater **(A)** and inter-rater **(B)** reliabilities for measurement of nerve cross-sectional area. CSA, cross-sectional area; IE, intra-epineurium method; OE, outside-epineurium method; ICC, Intraclass correlation coefficient; CI, confidence interval; UCL, upper confidence limit; LCL, lower confidence limit, SNBP, suprascapular nerve departing from the brachial plexus; SNMC, suprascapular nerve at the middle clavicular level; SNSS, suprascapular nerve at the floor of the supraspinatus fossa; SNIS, suprascapular nerve at the spinoglenoid notch of the infraspinatus fossa.

Regarding the CSA measurements across different sites, the intra-rater reliability (ICC) ranged from 0.555 to 0.884 (Figure 6A), whereas the inter-rater reliability (ICC) ranged from 0.394 to 0.785 (Figure 6B). The method defining the nerve CSA inside the epineurium was likely to have better reliability than the method measuring outside the epineurium.

### Univariate analysis of nerve CSA across different age and gender groups

Mean values and SD of nerve CSA in each subgroup are presented in Table 2. There was a trend of larger nerve CSAs in the male groups than those in the female groups. The aforementioned trend was less significant for the suprascapular nerve measured at the supraspinatus fossa and spinoglenoid notch using the outside-epineurium method. The nerve CSAs among the same sex but different age range were not significantly different across subgroups.

**Table 2:** Comparison of nerve cross-sectional area measurements (mm^2^) among different age and gender groups.

	**Age** ≥ **20 and** < **40**		**Age** ≥ **40 and** < **60**		**Age** ≥ **60**	
	**Men**	**Women**	**Men**	**Women**	**Men**	**Women**	***p*-value (overall)**
CSA (C5, IE)	8.37 ± 1.59^a^	6.38 ± 1.04^a^^d^^e^^f^	8.40 ± 1.30^d^	7.95 ± 0.72^e^	8.19 ± 1.59^f^	7.51 ± 0.91	<0.001
CSA (C6, IE)	9.14 ± 2.06	8.18 ± 1.54^d^	10.09 ± 1.44^d^^g^^h^	8.53 ± 0.89^g^	9.21 ± 1.27	8.33 ± 1.14^h^	<0.001
CSA (SNBP, IE)	2.04 ± 0.54^a^^b^^c^	1.58 ± 0.32^a^^d^^f^	2.10 ± 0.43^d^^g^^h^	1.52 ± 0.23^b^^g^^i^	2.14 ± 0.38^f^^i^^j^	1.37 ± 0.36^c^^h^^j^	<0.001
CSA (SNMC, IE)	2.07 ± 0.47^b^^c^	1.73 ± 0.61	2.03 ± 0.36^h^	1.63 ± 0.37^b^^i^	2.09 ± 0.43^i^^j^	1.42 ± 0.33^c^^h^^j^	<0.001
CSA (C5, OE)	15.63 ± 3.40^a^^c^	11.67 ± 1.76^a^^d^^e^^f^	16.17 ± 3.21^d^^h^	14.27 ± 2.38^e^	14.92 ± 2.77^f^	12.97 ± 1.76^c^^h^	<0.001
CSA (C6, OE)	16.87 ± 3.10	14.90 ± 2.49^d^	18.11 ± 3.09^d^	15.68 ± 2.36	16.97 ± 2.13	16.45 ± 2.16	0.004
CSA (SNBP, OE)	4.98 ± 1.89^c^	4.20 ± 1.20	4.78 ± 1.07^h^	4.05 ± 0.99	4.99 ± 0.96^j^	3.55 ± 0.86^c^^h^^j^	<0.001
CSA (SNMC, OE)	4.38 ± 0.66^c^	4.12 ± 1.40^k^	4.59 ± 069^h^	4.07 ± 0.98^l^	4.97 ± 0.82^j^	2.58 ± 1.48^c^^k^^h^^l^^j^	<0.001
CSA (SNSS, OE)	10.18 ± 2.29	9.34 ± 2.18	11.07 ± 2.80	9.31 ± 1.87	10.56 ± 1.31	9.73 ± 1.08	0.038
CSA (SNIS, OE)	10.99 ± 2.52	9.41 ± 9.62	9.62 ± 1.95	9.80 ± 1.67	9.35 ± 1.35	10.42 ± 1.40	0.048

*CSA, cross-sectional area; IE, inside-epineurium method; OE, outside-epineurium method; SNBP, suprascapular nerve departing from the brachial plexus; SNMC, suprascapular nerve at the mid-clavicular level; SNSS, suprascapular nerve at the floor of the supraspinatus fossa; SNIS, suprascapular nerve at the spinoglenoid notch of the infraspinatus fossa*.

***Annotation for post-hoc analysis of between-group difference***:

a * indicates significant between men (age ≥ 20 and < 40) and women (age ≥ 20 and < 40)*;

b * indicates significant between men (age ≥ 20 and < 40) and women (age ≥ 40 and < 60)*;

c * indicates significant between men (age ≥ 20 and < 40) and women (age ≥ 60)*;

d * indicates significant between women (age ≥ 20 and < 40) and men (age ≥ 40 and < 60)*;

e * indicates significant between women (age ≥ 20 and < 40) and women (age ≥ 40 and < 60)*;

f * indicates significant between women (age ≥ 20 and < 40) and men (age ≥ 60)*;

g * indicates significant between (age ≥ 40 and < 60)and women (age ≥ 40 and < 60)*;

h * indicates significant between men (age ≥ 40 and < 60) and women (age ≥ 60)*;

i * indicates significant between women (age ≥ 40 and < 60) and men (age ≥ 60)*;

j * indicates significant between women (age ≥ 60) and men (age ≥ 60)*;

k * indicates significant between women (age ≥ 20 and < 40) and women (age ≥ 60)*;

l * indicates significant between women (age ≥ 40 and < 60) and women (age ≥ 60)*.

### Multivariate analysis of factors associated with nerve CSA across different sites

The analysis derived from the GEE model revealed that female gender was negatively associated with the CSA values of the C5 and C6 nerve roots and the suprascapular nerve near the brachial plexus as measured by both methods (inside- and outside-epineurium). The significant association between sex and nerve CSAs diminished when measuring the suprascapular nerve distal to the mid-clavicular level. Age, sides (right/left) of the neural structures examined, body height, and weight were found to not be associated with the sizes of the C5 and C6 nerve roots or the suprascapular nerve at 4 target levels (Table 3).

**Table 3:** Multivariate analyses of the association of participants' characteristics with nerve cross sectional area using the generalized estimated equation.

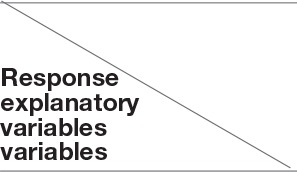	**CSA (C5, IE)**	**CSA (C6, IE)**	**CSA (SNBP, IE)**	**CSA (SNMC, IE)**	**CSA (C5, OE)**	**CSA (C6, OE)**	**CSA (SNBP, OE)**	**CSA (SNMC, OE)**	**CSA (SNSS, OE)**	**CSA (SNIS, OE)**
Age (year)	0.012 (*p =* 0.236)	0.004 (*p =* 0.677)	<0.001 (*p =* 0.974)	−0.001 (*p =* 0.820)	0.013 (*p =* 0.539)	0.015 (*p =* 0.343)	−0.004 (*p =* 0.586)	−0.005 (*p =* 0.424)	0.006 (*p =* 0.247)	−0.011 (*p =* 0.502)
Female gender (male as reference)	−0.885^*^ (*p =* 0.005)	−1.074^*^ (*p =* 0.011)	−0.532^*^ (*p* < 0.001)	−0.294 (*p =* 0.105)	−1.764^*^ (*p =* 0.034)	−2.221^*^ (*p =* 0.001)	−0.948^*^ (*p =* 0.030)	−0.518 (*p =* 0.263)	−1.020 (*p =* 0.063)	0.046 (*p =* 0.937)
Laterality (left as reference)	0.203 (*p =* 0.303)	−0.125 (*p =* 0.609)	−0.047 (*p =* 0.420)	−0.039 (*p =* 0.586)	0.213 (*p =* 0.581)	0.700 (*p =* 0.090)	−0.312 (*p =* 0.103)	−0.331 (*p =* 0.081)	−0.353 (*p =* 0.282)	−0.178 (*p =* 0.575)
Height (cm)	0.013 (*p =* 0.064)	−0.011 (*p =* 0.767)	0.007 (*p =* 0.379)	0.010 (*p =* 0.433)	0.072 (*p =* 0.265)	−0.029 (*p =* 0.640)	<0.001 (*p =* 0.986)	0.029 (*p =* 0.398)	0.013 (*p =* 0.762)	−0.045 (*p =* 0.168)
Weight (kg)	0.002 (*p =* 0.930)	0.015 (*p =* 0.467)	−0.001 (*p =* 0.911)	0.005 (*p =* 0.452)	0.005 (*p =* 0.923)	−0.019 (*p =* 0.637)	0.008 (*p =* 0.677)	0.017 (*p =* 0216)	0.001 (*p =* 0.968)	0.044 (*p =* 0.081)

*Annotation*:

* * indicates p < 0.05. CSA, cross-sectional area; IE, inside-epineurium method; OE, outside-epineurium method; SNBP, suprascapular nerve departing from the brachial plexus; SNMC, suprascapular nerve at the mid-clavicular level; SNSS, suprascapular nerve at the floor of the supraspinatus fossa; SNIS, suprascapular nerve at the spinoglenoid notch of the infraspinatus fossa*.

### Comparisons of the suprascapular nerve sizes at different levels

**Figure 7:**
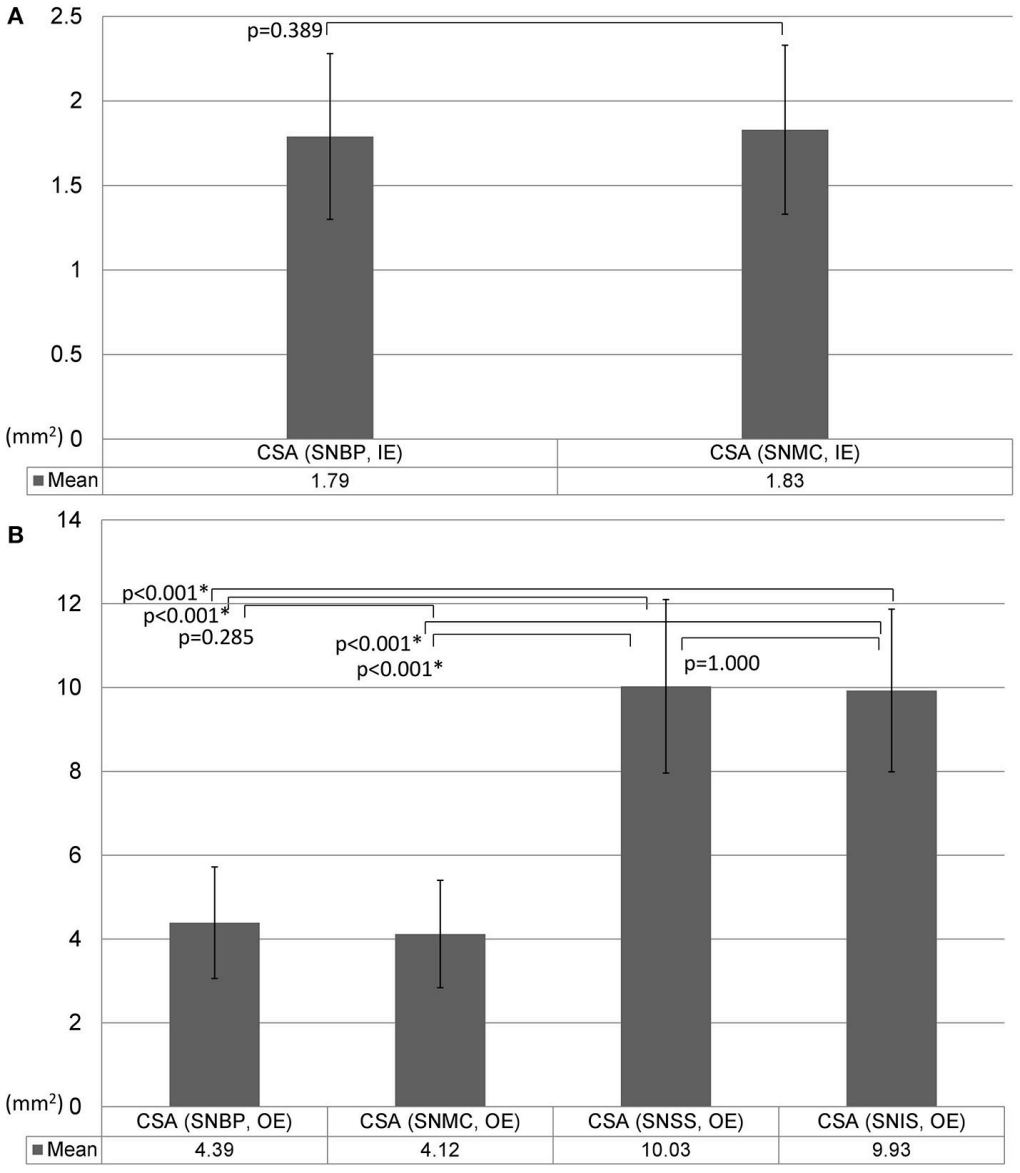
Comparison of the size of the suprascapular nerve measured by the inside-epineurium **(A)** and outside-epineurium **(B)** methods. CSA, cross-sectional area; IE, intra-epineurium method; OE, outside-epineurium method; SNBP, suprascapular nerve departing from the brachial plexus; SNMC, suprascapular nerve at the middle clavicular level; SNSS, suprascapular nerve at the floor of the supraspinatus fossa; SNIS, suprascapular nerve at the spinoglenoid notch of the infraspinatus fossa.

The suprascapular nerve CSA as measured by the inside-epineurium method was not significantly different between the brachial plexus level and the mid-clavicular region (Figure 7A). Using the outside-epineurium method, the values of distal nerve CSA (in the supraspinatus fossa and at the spinoglenoid notch) were significantly larger compared to the values at the proximal levels (near the brachial plexus and at the mid-clavicular region; Figure 7B).

## Discussion

This investigation resulted in several important findings. First, the inside-epineurium method was more reliable than the outside-epineurium approach for measurement of suprascapular nerve CSA due to its higher intra- and inter-rater reliability. Secondly, the CSA values of the C5 and C6 nerve roots and the suprascapular nerve near the brachial plexus were associated with gender difference, but not age, laterality, and body stature. Thirdly, employing the outside-epineurium method, the suprascapular nerve CSA is larger in its distal division than the portion at and proximal to the mid-clavicular line.

The suprascapular nerve, unlike the larger peripheral nerves such as median and sciatic nerves, has less degree of somatic organization. Therefore, its echotexture resembles the cervical nerve roots, which has a monofascicular pattern instead of a honeycomb appearance (23). Battaglia et al. measured the proximal segment of the suprascapular nerve for 33 asymptomatic subjects aged between 21 and 42 years and reported the mean nerve CSA to be 1.9 mm^2^ over the first rib and 2.0 mm^2^ at the distal clavicle (23). The details regarding CSA measurement (inside- or outside-epineurium) were lacking in the aforementioned study. In addition, no available literature reports the reference values of suprascapular nerve CSA from the brachial plexus level to infraspinatus fossa in different age and gender populations, as has been reported in this study.

In this study, we reported that the inside-epineurium method was more reliable than the outside-epineurium method in measuring the nerve CSA due to its higher intra- and inter-rater reliability. In recent years, with advancements in US technology, most high resolution US machines are able to delineate the ultrastructure of the peripheral nerves (24). The echotexture of nerve fascicles are hypoechoic, whereas the surrounding connective tissues like epineurium and perineurium appear hyperechoic (25). As the suprascapular nerve has a monofascicular pattern, the border between the nerve fascicle and epineurium is usually clearly defined; thus, it contributes to high reliability during CSA measurement. However, the epineurium is laminated and continuous with the mesoneurium, which is made up of loose areolar tissue (26, 27). The outer border of the epineurium is sometimes not well defined, especially when the nerve courses inside or passes through fasciae. Nevertheless, we investigated the CSA by using the outside-epineurium method for deeper nerves because the inner boundary of the epineurium was very hard to differentiate at the bottom of the supraspinatus and infraspinatus fossae.

There are multiple studies that report a correlation between the nerve CSA and participants' characteristics, such as age, sex, hand dominance, and body stature. As the aforementioned factors (e.g., men are usually taller and heavier than women) are inter-correlated and exhibit collinearity during multiple regression analysis, the association of nerve sizes with subjects' features varies across different reports. Regarding the upper extremity nerves, Sugimoto et al. studied 60 healthy Japanese adults and demonstrated that gender and wrist circumference are associated with CSA of the median and ulnar nerves at the non-entrapment sites (excluding the carpal, cubital and Guyon's canals) (14). In terms of the cervical regions, Huan et al. reported a trend of larger root sizes at the C5 and C6 levels in men than in women (28). In our study, the multivariate analysis demonstrates a negative association between female gender and the CSA values at the C5 and C6 nerve roots and the suprascapular nerve near the brachial plexus but not at its more distal levels. As the suprascapular nerve directly branches from the superior trunk, which is made up of the C5 and C6 nerve roots, it is rational to expect the size of the proximal suprascapular nerve to be in accordance with its root origins. Nevertheless, tracking a nerve to its peripheral portion, the nerve may become thinner or gives off its muscular and articular branches (7). Both the above-mentioned factors can impact the CSA measurements, thereby rendering gender difference to be a less significant concern in reporting CSA values for the distal suprascapular nerve.

Another important observation was that the size of the distal suprascapular nerve as measured by the outside-epineurium method was significantly larger than at its proximal portion. We propose 3 possible reasons that may contribute to this finding. First, the suprascapular nerve gives off the articular branch at the glenohumeral joint and muscular branch at the supraspinatus muscle in the supraspinatus fossa and muscular branch at the infraspinatus muscle in proximity to the spinoglenoid notch (7). As the high frequency transducer has limited resolution for structures that are located deep, it is challenging to employ US imaging to differentiate the nerve main stem from its branches (Figures 4C,D), all of which were therefore included in the CSA measurement. Second, the suprascapular nerve is accompanied by the suprascapular vessels in the supraspinatus and infraspinatus fossae. Although we had employed power Doppler imaging to identify adjacent vasculature, the small arterial and venous branches were still difficult to detect and may have falsely contributed to the enlarged nerve size. Third, the suprascapular nerve from the transverse scapular notch to the spinoglenoid notch is surrounded with variable amounts of perineural fat (Figure 4C). Some hyperechoic fatty tissues may have been included in the CSA measurements while employing the outside-epineurium method.

This study has two potential clinical implications. First, the CSA values of the most proximal suprascapular nerve are likely to be smaller in women than in men, with a difference of 0.5 mm^2^ by using the inside-epineurium method and a difference of 1 mm^2^ by using the outside-epineurium method (Table 2). Interpretation of pathological enlargement or atrophy of the suprascapular nerve may be based on a gender-specific reference, especially for observation in proximity to the brachial plexus. Secondly, the distal suprascapular nerve is not the same size as its proximal portion under US imaging. The clinicians should therefore employ segmental normal CSA ranges to diagnose suprascapular nerve disorders instead of comparing the target with other portions of the same nerve.

The study has several limitations that must be acknowledged. First, we only included Taiwanese participants. Whether the study's result can be applied on other ethnic group remains uncertain. Second, only one rater participated in the majority of the measurements although the pre-examination inter-rater reliability seemed satisfactory. A systematic error could possibly happen when nearly all the evaluations were done by a single investigator. Third, we did not use the cadaveric models to validate our approaches for quantifying nerve CSA. Future prospective studies are required to compare the nerve size measured by US with those (with/without epineurium) dissected on cadavers as well as to examine the diagnostic utility of CSA normal values as reported in this study on patients with painful shoulders.

## Conclusions

This US study reports the CSA reference values of the suprascapular nerve from its origin to the spinoglenoid notch. The inside-epineurium method has better reliability; however, the outside-epineurium method is still required for assessment of the distal suprascapular nerve. Gender difference in CSA values should be taken into account during evaluation of the most proximal nerve segment. Employing the outside-epineurium method, the distal suprascapular nerve is estimated to be larger than its proximal portion and this segmental discrepancy should not be misinterpreted as pathology.

## Author contributions

K-VC conceived and supervised this work. W-TW, KM, ON, C-PL, and LÖ carried out the research, implementation, validation, and analysis with input from K-VC. W-TW wrote the manuscript with critical feedback from K-VC.

### Conflict of interest statement

The authors declare that the research was conducted in the absence of any commercial or financial relationships that could be construed as a potential conflict of interest.
